# High-density genetic mapping identified QTLs for anaerobic germination tolerance in rice

**DOI:** 10.3389/fpls.2022.1076600

**Published:** 2022-12-23

**Authors:** Wenhua Liang, Hongyang Du, Bingwen Pang, Junjie Cheng, Bing He, Fengqin Hu, Yuanda Lv, Yadong Zhang

**Affiliations:** ^1^ Institute of Food Crops, Jiangsu Academy of Agricultural Sciences, Nanjing, China; ^2^ Excellence and Innovation Center, Jiangsu Academy of Agricultural, Sciences, Nanjing, China; ^3^ Key Laboratory of Rice Genetic Breeding of Anhui Province, Rice Research Institute, Anhui Academy of Agricultural Science, Hefei, China

**Keywords:** Rice, High-density Genetic Map, Anaerobic Germination, QTL, RNA-seq

## Abstract

The tolerance of rice anaerobic germination (AG) is the main limiting factor for direct seeding application, yet the genetics mechanism is still in its infancy. In the study, recombinant inbred lines population of TD70 *Japonica* cultivar and Kasalath Indica cultivar, was employed to construct a high-density genetic map by whole genome re-sequencing. As a result, a genetic map containing 12,328 bin-markers was constructed and a total of 50 QTLs were then detected for CL(coleoptile length), CD (coleoptile diameter), CSA (coleoptile surface area) and CV (coleoptile volume) related traits in the two stages of anaerobic treatment using complete interval mapping method (inclusive composite interval mapping, ICIM). Among the four traits associated with coleoptile, coleoptile volume had the largest number of QTLs (17), followed by coleoptile diameter (16), and coleoptile length had 5 QTLs. These QTLs could explain phenotypic contribution rates ranging from 0.34% to 11.17% and LOD values ranging from 2.52 to 11.57. Combined with transcriptome analysis, 31 candidate genes were identified. Furthermore, 12 stable QTLs were used to detect the aggregation effect analysis. Besides, It was found that individuals with more aggregation synergistic alleles had higher phenotypic values in different environments. Totally, high-density genetic map, QTL mapping and aggregation effect analysis of different loci related to the anaerobic germination of rice seeds were conducted to lay a foundation for the fine mapping of related genes in subsequent assisted breeding.

## Introduction

Rice is an essential food crop for billions of people. However, rice transplanting is costly for labor and water resources. Along with the development of the economy and restrained water and labor supply, rice planting and production systems are gradually transforming. Direct seeding in rice cultivation (DSR) is increasingly adopted to save labor and production cost (Mahender et al., 2015). However, direct seeding in actual production conditions also has disadvantages, such as direct seeding in water. In cold conditions, hypoxia, competition with weeds, and other adverse factors make seeds have poor germination, and seedlings wilt or die (Farooq et al., 2011). Hypoxia stress, accompanied by the germination and seedling stage, is the primary environmental stress for direct water seeding, which limits the germination rate and seedling growth of rice, thus affecting the yield. Therefore, the tolerance of rice anaerobic germination (AG) is the main limiting factor for the large-scale development of direct seeding. Revealing the genetic and molecular mechanisms of AG tolerance under flooding conditions and cultivating rice varieties with excellent AG resistance are important ways to effectively solve the limitations of direct seeding in rice production.

The tolerance of rice seedlings to hypoxia stress during germination is a complex quantitative trait controlled by multiple gene loci. In previous studies, various genetic populations have been used for linkage and association mapping to identify many QTLs related to seed germination and early seedling tolerance to hypoxia stress (Angaji, 2008; Angaji et al., 2010; Septiningsih et al., 2013; Baltazar et al., 2014; Hsu and Tung, 2015; Baltazar et al., 2019; Yang et al., 2019; Ignacio et al., 2021; Liu et al., 2021). Using the Kinmaze/DV85 recombinant inbred line population, Jiang et al. identified five QTLs on chromosomes 1, 2, 5 and 7, respectively, which could explain 10.5% - 19.6% of the phenotypic variation. In another study, Jiang et al. (2006) identified two QTLs possibly related to AG potential by using an F_2_ segregation population constructed by *Japonica* cultivar USSR5 and *Indica* cultivar N22, located on chromosomes 5 and 11, respectively, with the phenotypic variation of 11% - 15.5% (Jiang et al., 2006). Six QTLs related to AG were detected in the F_2:3_ mapping population constructed by IR42 and MR; Among them, *qAG7.1* (AG2) is the most effective QTL on chromosome 7, with a LOD value of 14.5 and a phenotypic variation of 31.7% (Septiningsih et al., 2013). Baltazar et al. identified four QTLs related to anaerobic germination using the F_2:3_ population of IR64/Kharsu, including three on chromosome 7 and one on chromosome 3, with a LOD value of 5.7-7.7, which could explain 8.1% - 12.6% of the phenotypic variation (Baltazar et al., 2019). In another study, a new site *qACE3.1* was identified in the chromosome segment substitution lines (CSSLs) population constructed by IR64 and Koshihikari under anaerobic conditions, which can promote the elongation of coleoptiles under anaerobic conditions (Nishimura et al., 2020).

Recently, the BC_2_F_2_ population was constructed with IR64 (*Japonica* cultivar) as the recurrent parent and Kho Hlan on (KHO, *Japonica* cultivar) as the receptor, and five possible QTLs related to flooding tolerance were detected. Among them, *qAG-9-2* (AG1 for short) is a major QTL located on the long arm of chromosome 9, with a LOD value of 20.3, which could explain 33.5% of the phenotypic variation (Angaji et al., 2010). Subsequently, the QTL was cloned as *OsTPP7* gene, encoding a trehalose-6-phosphate phosphatase. It is functional in the metabolism of trehalose-6-phosphate, and the expression of the gene could increase the pool capacity in heterotrophic tissues, and thus the utilization of starch, which resulted in promoting seed germination and coleoptile growth and enhancing hypoxia tolerance during germination (Kretzschmar et al., 2015). This QTL has been used for crop improvement and has been successfully verified (Chamara et al., 2018; Mondal et al., 2020; Mondal et al., 2020). These results provided important genetic information for improving the potential of AG and making it serve molecular breeding.

SNP genotyping by next-generation sequencing technology (NGS) improves the accuracy of genetic mapping and dramatically promotes the mining and identification of key genes. Through GWAS analysis and QTL mapping, a hexokinase gene (HXK6, LOC_Os01g53930) was identified in 144 RILs of Nipponbare/IR64. Studies showed that this gene was related to the elongation of rice coleoptiles under anaerobic conditions (Hsu and Tung, 2015). A total of 25 QTLs related to anaerobic germination were identified in YZX (Indica rice) and 02428 (*Japonica* cultivar) RIL populations using a high-density genetic map containing 2711 bin markers. 13 stable QTLs were further identified from them, and 88 differentially expressed bases were screened by combining transcriptome (Yang et al., 2019). A high-density map containing 1070 bin markers was constructed using 131 introgression lines with 93-11 as receptors and W2014 as donors. One QTL, *qAGP1* and *qAGP3* were detected on chromosomes 1 and 3, which could explain 15% of the phenotypic variation. These two QTLs improved hypoxia tolerance by increasing coleoptile length during rice germination (Liu et al., 2021). Although many QTLs related to hypoxia tolerance in germination have been identified, only a few use substitution lines or near-isogenic lines to evaluate their effects on rice seedling growth, and the molecular mechanism of anaerobic tolerance needs further study.

Traditional QTL mapping and molecular marker genotyping are time-consuming and laborious (Chen et al., 2014). Simple sequence repeats (SSRs) and other low throughput molecular markers are often used to construct rice QTL linkage maps and QTL analysis. Most of them have low density and cannot provide the precise location of QTLs controlling interesting traits (Matsunami et al., 2011). SNP occurs in almost all population individuals with high density. The new generation of sequencing technology can facilitate directly obtaining population single nucleotide polymorphism (SNP) markers for genotyping (Kumar et al., 2012). Previous studies in rice have proved that the improvement of the quality and resolution of high-density genetic maps based on SNP markers has greatly improved the efficiency and accuracy of QTL mapping (Huang et al., 2009; Gao et al., 2013; Wang et al., 2018; Yang et al., 2021).

In this study, we demonstrated the difference in coleoptile phenotype in RILs populations during AG of two distinct genotypes. A high-density genetic map was constructed by whole genome resequencing. QTLs linked with coleoptile phenotype were identified at two stages of anaerobic germination. Furthermore, we performed transcriptome analysis to identify differentially expressed genes (DEG) in the localization regions related to AG tolerance, which could provide valuable information for verifying candidate genes and analyzing genetic and molecular mechanisms affecting seed AG tolerance. At the same time, it provides a reference for molecular marker-assisted selection of rice varieties suitable for direct seeding.

## Materials and methods

F_1_ was generated by crossing between *Japonica* cultivar TD70 and *Indica* cultivar Kasalath, and a population F_10_ containing 186 recombinant inbred lines (RIL) was constructed by the single seed descent method. *Japonica* cultivar TD70 is the offspring of Tianegu///9520//(72-496/Suyunuo), which is a stable line independently created by the research team. Kasalath is a conventional *Indica* rice variety in India, which is provided by the germplasm resources protection and utilization platform of Jiangsu Province. The experimental materials were planted in the experimental field of Jiangsu Academy of Agricultural Sciences, China. Each RIL or parent was planted in 4 rows, with ten plants in each row, 13.5cm plant spacing, 26.5cm row spacing, and conventional cultivation and management. Considering the influence of seed maturity on AG, the plants in the middle of each plot are harvested after the seeds are fully mature (50 days after heading). The harvested seeds were dried in a 40 °C hot-air dryer for 6 days and then stored at - 20 °C.

A total of 30 plump and uniform seeds were selected from three independent individual plants harvested in each experimental plot and placed in an oven at 50 °C for seven days to relieve seed dormancy. Then, the seeds were disinfected with 6% sodium hypochlorite for 30 min, and rinsed with sterile water 6-7 times. Take five sterile seeds and place them in a glass tube with a height of 8 cm. Add sterile water with a depth of 5 cm to create a hypoxic environment (Hsu and Tung, 2015). Then put it into a 28 °C incubator for dark cultivation. On the 4-6th day of treatment, the coleoptile length (CL), coleoptile diameter (CD), coleoptile surface area (CSA) and coleoptile volume (CV) were measured using cloud platform (www.irootanalysis.cn/index/indexHome). Three replicates were analyzed for each strain. SPSS (version 22) and Excel software were used for statistical data analysis.

### DNA extraction, sequencing, typing and SNP identification

DNA was isolated from the leaves of two parents and 186 F_10_ RILs at the tillering stage by the CTAB method. DNA quality was detected by agarose gel electrophoresis and nanodrop nd1000 spectrophotometer. In this study, the genome re-sequencing depth of parents TD70 and Kasalath is ~20×. The average sequencing depth of RILs is 10×. Sequencing was carried out with Illumina sequencing platform NovaSeq 6000, and the sequencing mode is paid end 150 bp. After low-quality base filtration of sequenced reads, they were compared to the reference genome using BWA software (Li and Durbin, 2009) (http://rapdb.dna.affrc.go.jp/ , IRGSP-1.0 version ). Then, SAMtools (Li et al., 2009) and PICARD (http://picard.sourceforge.net ) were used to remove redundancy. GATK (McKenna et al, 2010) was performed for calling SNP and genotype identification.

### High-density genetic map construction and QTL analysis

The resequencing of 186 RIL lines from TD70/Kasalath was performed for SNP identification, and high-quality, biallelic homozygous loci were obtained. To avoid false positive SNP genotyping in the population, Huang et al. (2009) used the sliding window method and made appropriate modifications to evaluate a group of continuous SNPs and perform genotyping. The genotype of RILs is split into a series of recombinant bins according to recombination breakpoints. The sliding-window SNP number of these bins is 15 and adjacent Bins within 200 kb are merged. The bin genotype of the transition region between two different genotype blocks is set as the missing data. The abnormal separation mark showed that the partial separation (P<0.01) was discarded.

QTL IciMapping v4.1 software (Meng et al., 2015) was used to construct the genetic map using the Kosambi map method and analyze the four traits related to coleoptiles on the 4-6th days after anaerobic stress treatment of the recombinant inbred lines. The inclusive composite interval mapping (ICIM-ADD) method was used for QTL identification. The logarithm of odds (LOD) score threshold was set to 2.5. Loci with LOD ≥ 2.5 are deemed candidate QTLs associated with the trait. QTL naming refers to McCuch’s method (McCouch, 2008).

### RNA isolation, sequencing and expression profile analysis

In order to obtain expression profiles and differentially expressed genes related to anaerobic germination, the seeds of parents TD70 and Kasalath were taken for RNA Seq experiments with/without anaerobic treatment, respectively. The seeds were rinsed with double-distilled water, and then 5-6 seeds were taken from each sample, and three biological replicates were set, and thus a total of 12 samples were taken. These samples were immediately put into liquid nitrogen for quick freezing and stored at - 80 °C for RNA isolation. The purity, concentration and integrity of the total RNA isolated were evaluated, and RNA seq library construction and sequencing were referred to the previous process (Liang et al., 2022).

The Fastp program (Chen et al., 2018) was engaged to remove the adapter, low-quality sequencing tail, and then the Hiast2 program (Pertea et al., 2016) was used for mapping reads to the reference genome (http://rapdb.dna.affrc.go.jp/ , IRGSP-1.0 version). SAMtools (Li et al., 2009) were used for converting format. The expression abundance of transcripts was calculated based on fragments per kilobase of transcript per million fragments mapped (FPKM). The transcripts with a maximum FPKM value of less than 1 in multiple samples were eliminated as no expression or false positive transcripts (Trapnell et al., 2012). Differential expression genes were screened by the DESeq2 software package (Anders and Huber, 2010), with screening criteria of Log2 (fold change) ≥ 1 and q-value<0.05.

## Results

### Responses of parents and RIL opulations to AG at the germination stage

**Figure 1:**
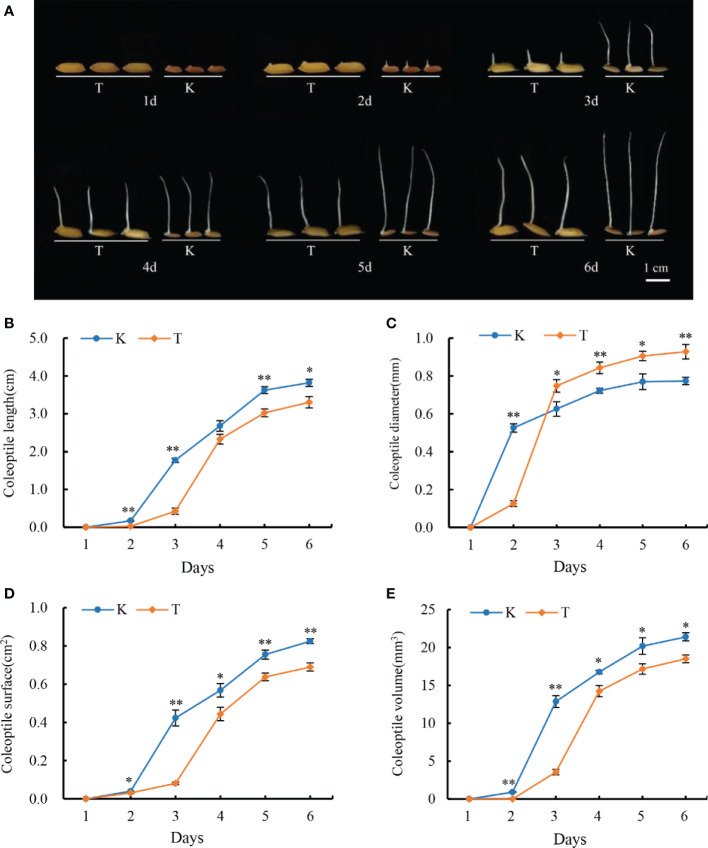
Dynamic of TD70 and Kasalath coleoptile developments under anaerobic conditions **(A)**. The phenotypes of two parents 1-6 days under anaerobic conditions; **(B–E)** represent the change of coleoptile length, diameter, surface area, and volume 1-6d under anaerobic conditions, respectively. K, Kasalath; T, TD70; * and ** represent the significant *p*<0.05 and *p*< 0.01, respectively.

The faster the rice coleoptiles elongate, the sooner the seedlings escape from an anoxic environment and increase the survival chances of rice. Therefore, rice coleoptile is a classic tissue for studying AG tolerance (Narsai et al., 2015). In this study, we investigated the dynamic changes of coleoptiles of two parents under anaerobic conditions; Significant variation in coleoptile traits was observed between TD70 and Kasalath, which were the parents of both (**Figure 1**).

After one day of treatment, Kasalath seeds began to germinate, and obvious coleoptiles bulged, while TD70 showed no significant change. On the second day, Kasalath showed obviously elongated coleoptiles, while TD70 seeds had just begun to germinate, and prominent coleoptiles emerged, which showed that Kasalath seeds germinated faster than those of TD70. The development of Kasalath coleoptile was occurred mainly from the second to the fifth day of treatment, while the growth of TD70 coleoptile mainly occurred on the third day. On the second day of treatment, the growth rate of coleoptile length (CL) of Kasalath was significantly higher than that of TD70, and the difference was very significant; However, the CL of TD70 increased rapidly from the third day to the fourth day of the treatment, and there was no significant difference between them on the fourth day. Then, on the fifth and sixth days, the CL length of kasalath was significantly longer than that of TD70, and the difference was significant. It could be seen that, on average, the CL elongation speed of Kasalath was faster (**Figures 1A, B**). From the third day of treatment, the CSA and CV of Kasalath were significantly higher than those of TD70. However, from the third day, the CD of TD70 was significantly larger than that of Kasalath, and the difference was significant or extremely significant until the sixth day.

Thus, the phenomenon stated above indicated that the coleoptile development of Kasalath mainly occurred on the first and second days of treatment. In contrast, TD70 mainly occurred on the third day (**Figure 1**). Thus, the four agronomy traits with significant or extremely significant differences in AG anaerobic treatment between the parents TD70 and Kasalath indicate that there were great genetic differences between them, on which QTLs mapping was conductive based.

**Figure 2:**
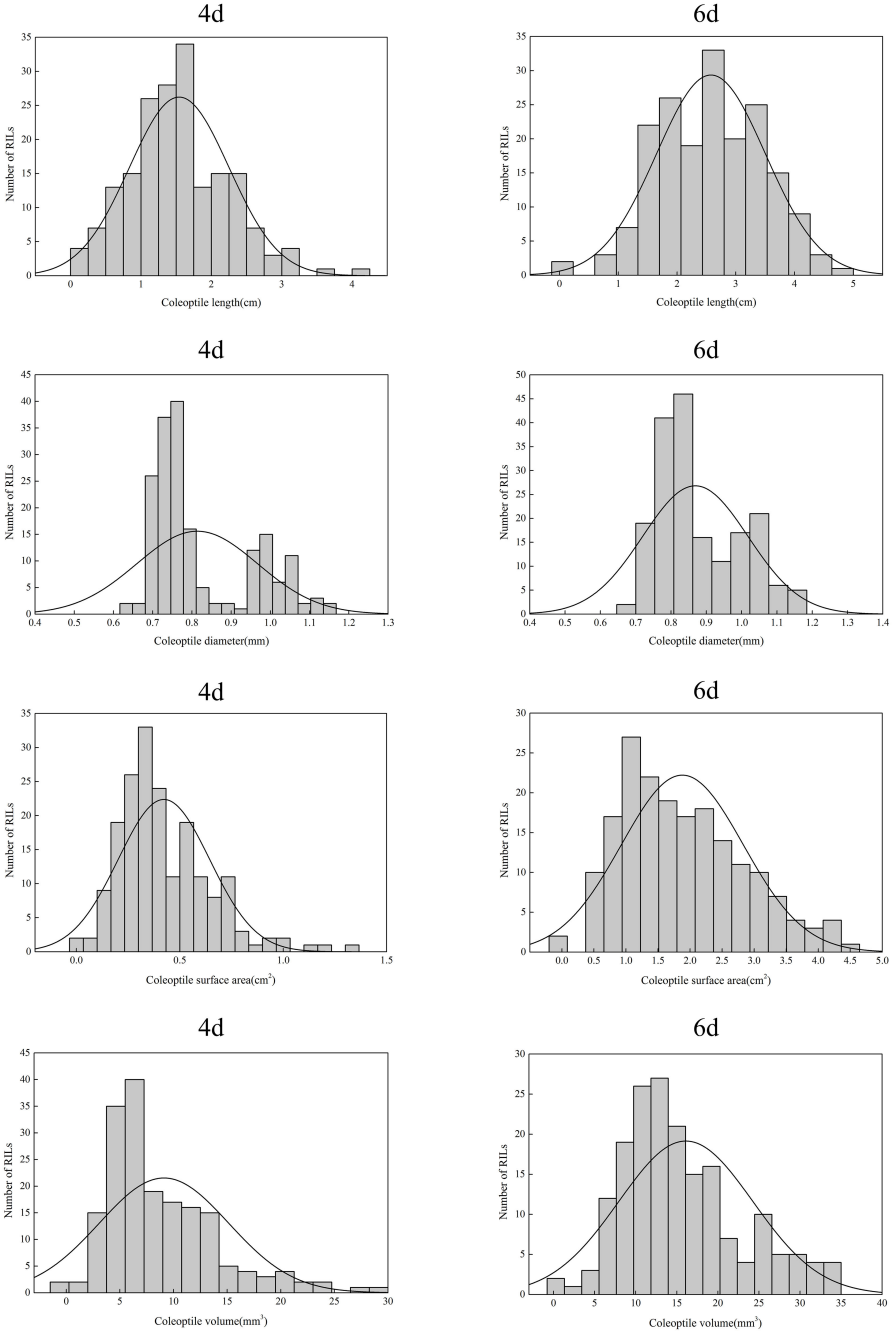
The distribution of four coleoptile phenotype traits in the RIL population.

The four traits of the coleoptile phenotype showed different segregation in the RIL population (**Figure 2**). The coefficient of variation (CV) on the fourth day of treatment ranged from 18.92% to 66.19%; The coefficient of variation at six days was 17.09% - 52.21%. Moreover, these traits show transgressive segregation in variated degrees. All characters are continuous unimodal distribution, and the absolute skewness value is close to 0 in each character, indicating that all these characters conform to the normal distribution and are controlled by multiple micro genes (**Table 1**).

**Table 1:** Phenotypes of TD70, Kasalath and RIL population of offspring at two germination stages under anaerobic conditions.

Trait	Days	Parents		RIL population				
		TD70	Kasalath	Mean	Range	Skewness	Kurtosis	CV^a^ (%)
CL(cm)	4	2.32 ± 0.12	2.68 ± 0.14	1.54	0.13-4.23	0.54	0.75	45.84
	6	2.88 ± 0.08	3.81 ± 0.11	2.57	0.76-5.19	0.01	-0.15	35.91
CD(mm)	4	0.84 ± 0.03	0.72 ± 0.01	0.81	0.64-1.15	-0.95	6.92	18.92
	6	0.93 ± 0.04	0.77 ± 0.04	0.87	0.68-1.18	-1.73	10.91	17.09
CSA(cm^2^)	4	0.44 ± 0.04	0.57 ± 0.04	0.42	0.09-1.34	1.13	1.97	52.21
	6	0.69 ± 0.02	0.82 ± 0.01	1.88	0.42-4.56	0.54	-0.37	50.55
CV(mm^3^)	4	14.23 ± 0.73	16.75 ± 0.73	9.13	1.70-34.67	1.76	3.84	66.19
	6	19.33 ± 0.57	21.41 ± 0.53	16.12	3.13-45.78	0.95	0.72	50.47

CL, coleoptile length; CD, coleoptile diameter; CSA, coleoptile surface area; CV, coleoptile volume. Parent refers to the mean ± standard deviation (SD) of the parents; CV^a^, coefficient of variation.

### Population genotyping and binmap construction

This study obtained 1,304 Gb high-quality sequencing data from illumina paired-end sequencing. The average sequencing depth is 18.8×. Among them, ~97.25% of the reads could be uniquely mapped to the reference genome. These SNP sites of RIL individuals were identified, and SNPs with depth >5 were retained. After filtering exceptions, 1,344,770 high-quality SNPs were finally used for subsequent analysis of recombination events and construct binmap, and the map contains 12,328 bin markers. All bin markers are evenly distributed on 12 chromosomes. The number of markers on chromosome 1 was the largest, 148,582; chromosome 9 has the least markers, 87,301(**Table S2**).

### Identification of AG-associated QTLs

Using the inclusive composite interval mapping (ICIM) method, 50 QTL loci associated with CL, CD, CSA and CV were identified in two stages of anaerobic treatment. These QTLs are distributed on all chromosomes except chromosome 9. There were as many as 10 loci distributed on chromosome 4, followed by 7 loci detected on chromosome 8 and 12, respectively; only one epistasis was detected on chromosome 2. Among the four traits related to coleoptile, the number of QTLs detected by coleoptile volume was the largest (17), followed by coleoptile diameter (16), and there were 5 QTLs associated with coleoptile length. These QTLs can explain phenotypic contribution rates ranging from 0.34% to 11.17% and LOD values ranging from 2.52 to 11.57. Some QTL loci in this study showed a positive additive effect and some negative additive effect, suggesting that both parents contributed favorable alleles.

**Figure 3:**
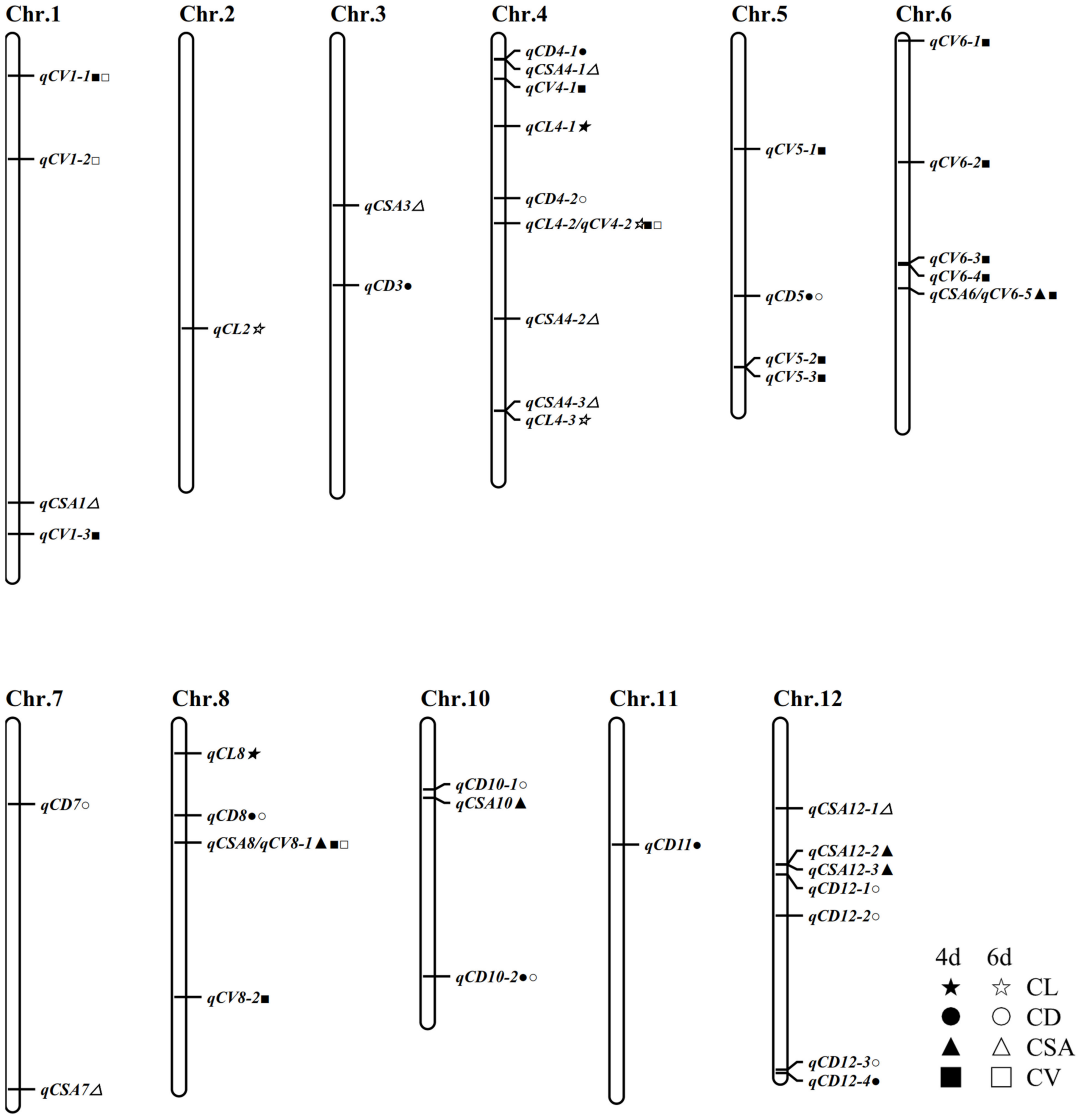
Chromosome distribution of these stable QTLs.

There are 5 QTLs associated with CL. The contribution rate of a single QTL ranged from 4.85% - 10.99%. There were 12 loci associated with CSA, and the phenotypic contribution rate was between 1.40% to 9.39%. Sixteen QTLs were associated with CD, which could explain the phenotypic variation of 2.05% - 11.17%. Seventeen QTLs were associated with CVs, with phenotypic contribution rates ranging from 0.34% to 9.37%. QTLs showing physical-position overlap were considered as mere one QTL site. Finally, 42 QTLs were obtained in two stages of anaerobic germination. It was remarkable that five QTLs were detected in two germination stages: *qCD5*, *qCD8*, *qCV1-1*, *qCD10-2* and *qCV8-1*. Three loci were detected simultaneously by two different traits, of which *qCSA6* and *qCV6-5* overlapped as just one locus, and as well as *qCV4-2* and *qCL4-2*, *qCSA8* and *qCV8-1*, respectively; and they affected CL, CSA and CV (**Table 2**; **Figure 3**).

**Table 2:** QTLs associated with coleoptile traits during two periods of hypoxic germination.

QTL	Days	Chr.	Marker interval	Physical interval	LOD	PVE(%)	Add	Known locus
qCV1-1	4	1	Bin117-Bin118	2890073-2949395	10.45	0.55	-6.77	
qCV1-1	6	1			3.39	7.66	-6.30	
qCV1-2	6	1	Bin329-Bin330	9616532-9707372	3.19	1.62	-2.20	Liu et al., 2021
qCV1-3	4	1	Bin1250-Bin1251	39821205-39855940	8.20	0.55	-7.10	
qCSA1	6	1	Bin1193-Bin1194	37290282-37308515	4.48	4.34	-0.33	
qCL2	6	2	Bin2038-Bin2039	23247409-23313064	2.64	9.13	0.42	
qCSA3	6	3	Bin2655-Bin2656	13334166-13418232	4.24	9.39	-0.85	
qCD3	4	3	Bin2856-Bin2857	19769142-19898047	2.52	5.59	-0.05	
qCD4-1	4	4	Bin3370-Bin3371	1543556-1562998	2.65	3.64	-0.07	
qCSA4-1	6	4	Bin3374-Bin3375	1600851-1645142	3.04	2.90	0.32	
qCV4-1	4	4	Bin3454-Bin3455	3154153-3191419	5.10	0.54	-6.76	
qCL4-1	4	4	Bin3589-Bin3590	6944055-6998203	3.30	6.88	0.35	
qCD4-2	6	4	Bin3725-Bin3726	12757286-12823893	3.02	2.05	-0.04	
qCL4-2	6	4	Bin3788-Bin3789	14780687-14808655	3.15	5.59	0.25	Yang et al., 2021
qCV4-2					3.24	1.75	2.18	
qCSA4-2	6	4	Bin4035-Bin4036	22473081-22499182	3.42	3.31	0.25	Yang et al., 2019
qCSA4-3	6	4	Bin4274-Bin4275	29885854-29920615	8.28	9.21	0.45	
qCL4-3	6	4	Bin4275-Bin4276	29893048-29933483	2.80	4.85	0.28	
qCV5-1	4	5	Bin4778-Bin4779	8798783-8858855	8.14	0.55	-6.64	
qCV5-2	4	5	Bin5336-Bin5337	26369420-26471049	7.20	0.55	-6.96	
qCV5-3	4	5	Bin5337-Bin5338	26385407-26518791	4.34	0.51	-6.93	
qCD5	4	5	Bin5167-Bin5168	20629588-20770108	5.54	11.17	0.16	
qCD5	6	5			8.14	7.02	0.17	
qCSA6	4	6	Bin6167-Bin6168	20022371-20096670	2.57	2.24	-0.17	
qCV6-5	4	6			7.54	0.54	-6.65	
qCV6-1	4	6	Bin5442-Bin5443	80687-133609	7.51	0.55	-7.13	
qCV6-2	4	6	Bin5787-Bin5788	9858384-9898904	4.90	0.34	-11.06	
qCV6-3	4	6	Bin6097-Bin6098	17995332-18029267	7.98	0.55	-6.97	
qCV6-4	4	6	Bin6106-Bin6107	18133500-18186833	4.01	0.55	-6.24	
qCD7	6	7	Bin6768-Bin6769	6187353-6232165	2.98	2.15	-0.04	
qCSA7	6	7	Bin7527-Bin7528	28442520-28483618	2.76	3.62	0.24	Hsu and Tung, 2015
qCL8	4	8	Bin7664-Bin7665	2229665-2259731	5.82	10.99	0.26	
qCD8	4	8	Bin7843-Bin7844	7055955-7089043	6.82	8.89	0.37	
qCD8	6	8			8.71	5.25	0.38	
qCSA8	4	8	Bin7941-Bin7942	9196326-9222900	3.89	2.02	-0.21	
qCV8-1	4	8			11.57	0.55	-7.47	
qCV8-1	6	8			2.79	9.37	-6.48	
qCV8-2	4	8	Bin8412-Bin8413	21238647-21272554	6.14	0.53	-7.69	Yang et al., 2021
qCD10-1	6	10	Bin9581-Bin9582	5035933-5086795	5.48	6.86	0.32	
qCD10-2	4	10	Bin10082-Bin10083	19629777-19696813	3.09	4.61	-0.04	
qCD10-2	6	10			3.80	2.68	-0.04	
qCSA10	4	10	Bin9611-Bin9612	5704076-5752096	3.54	1.40	-0.37	
qCD11	4	11	Bin10524-Bin10525	9342548-9405943	2.59	3.48	-0.05	
qCSA12-1	6	12	Bin11505-Bin11506	6519288-6539105	3.62	4.40	-0.28	
qCSA12-2	4	12	Bin11694-Bin11695	10902983-10925501	2.71	2.09	-0.16	
qCSA12-3	4	12	Bin11695-Bin11696	10910092-10952995	2.62	2.02	-0.16	
qCD12-1	6	12	Bin11736-Bin11737	11680541-11708141	3.36	3.47	0.09	
qCD12-2	6	12	Bin11913-Bin11914	14891363-14927725	3.51	4.38	0.14	
qCD12-3	6	12	Bin12307-Bin12308	26918303-26957012	4.79	3.53	-0.05	Hsu and Tung, 2015; Yang et al., 2019
qCD12-4	4	12	Bin12318-Bin12319	27174700-27205921	3.90	5.82	-0.04	

ADD, Additive effect, a positive value indicates the superiority of japonica TD70.

We found 6 out of the 42 loci have been previously reported (**Table 2**). The location of *qCV1-2* overlaps with the physical interval of which *qAGP1* (Liu et al., 2021). *QCL4-2/qCV4-2* and *qCV8-2* are in the same physical regions as *qSFW-4* and *qRD-8*, respectively (Yang et al., 2021). The physical area of *qCSA4-2* is consistent with that of *qCL-4-1*; The physical location of *qCD12-3* coincides with that of *qAG12/qCSA-12-2* (Hsu and Tung, 2015; Yang et al., 2019). *qCSA7* locates on chromosome 7, and its physical location in a region from where many QTLs related to anaerobic germination were found (Ling et al., 2004; Angaji et al., 2010; Septiningsih et al., 2013; Hsu and Tung, 2015). The results of the above report indicate the accuracy of our mapping result.

### Pyramiding effects of stable QTLs

In the present study, three kinds of QTLs were defined as stable QTLs, including overlapping QTLs associated with multiple traits; the loci detected in both periods and the loci colocalized with reported QTL loci. There were thus 12 stable loci discovered in this study, and they were associated with 20 QTLs (**Table 3**). To further clarify the effects of these QTLs, we summarized the phenotypic differences between two alleles at each locus in the RIL population.

**Table 3:** Identified 12 stable QTL loci.

Loc	QTL	Chr.	Marker interval	Physical interval
Loc1	qCV1-1(4d/6d)	1	Bin117-Bin118	2890073-2949395
Loc2	qCV1-2(6d)	1	Bin329-Bin330	9616532-9707372
Loc3	qCL4-2(6d);qCV4-2(6d)	4	Bin3788-Bin3789	14780687-14808655
Loc4	qCSA4-2(6d)	4	Bin4035-Bin4036	22473081-22499182
Loc5	qCD5(4d/6d)	5	Bin5167-Bin5168	20629588-20770108
Loc6	qCSA6(4d);qCV6-5(4d)	6	Bin6167-Bin6168	20022371-20096670
Loc7	qCSA7(6d)	7	Bin7527-Bin7528	28442520-28483618
Loc8	qCD8(4d/6d)	8	Bin7843-Bin7844	7055955-7089043
Loc9	qCSA8(4d);qCV8-1(4d/6d)	8	Bin7941-Bin7942	9196326-9222900
Loc10	qCV8-2(4d)	8	Bin8412-Bin8413	21238647-21272554
Loc11	qCD10-2(4d/6d)	10	Bin10082-Bin10083	19629777-19696813
Loc12	qCD12-3(6d)	12	Bin12307-Bin12308	26918303-26957012

Firstly, the RIL population was divided into TD70 type and Kasalath type according to the genotype of each marker site, and then the corresponding traits were compared between the individuals of the two types. The result showed that these 8 of 12 loci significantly affected coleoptile traits. The average value of the traits in the individuals with excellent alleles was higher than in those without excellent alleles. For instance, *qCL4-2*/*qCV4-2*, the maximum absolute difference in coleoptile length between the two alleles was 0.36 cm, and the volume difference was 2.44 mm^3^ which was highly significant. The maximum difference in coleoptile volume of *qCV1-2* between the two alleles was 1.91 mm^3^, and the maximum absolute difference of coleoptile length was 0.31 cm, which was significant. This shows that these 8 loci were reliable and responsible for phenotypic variation. The other four loci were associated with CV and CSA. The average phenotypes of individuals with excellent alleles were higher than those of individuals without (**Supplementary Table S3**).

**Figure 4:**
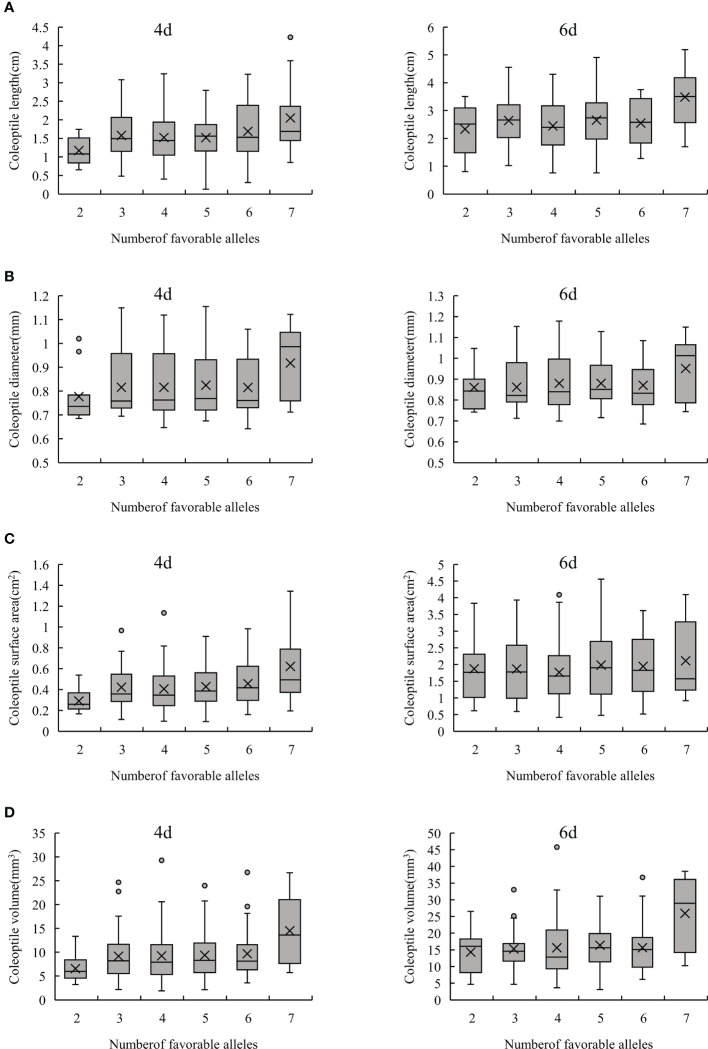
Phenotypic analysis of favorable alleles aggregation. **(A–D)** represents CL, CD, CSA and CV, respectively.

In order to further confirm the result shown above, the effects of these excellent alleles were evaluated by polymerization of different loci. If the interaction between these 12 stable loci were not considered, the more excellent alleles aggregated in one individual would result in a better phenotypic value of these traits. In this study, most of the lines in the RIL population contain 3-7 favorable alleles, and their corresponding phenotypic value increased along with the aggregation of favorable alleles (**Figure 4**). In this study, 12 lines containing 7 favorable alleles were obtained. Under hypoxia conditions, the length of coleoptiles grew rapidly, and their surface area and volume were also larger. Hence their AG tolerance was strong (**Figure 4**; **Supplementary Table S4**). These results indicate that aggregation of favorable alleles could improve AG tolerance.

### Candidate genes associated with AG

**Figure 5:**
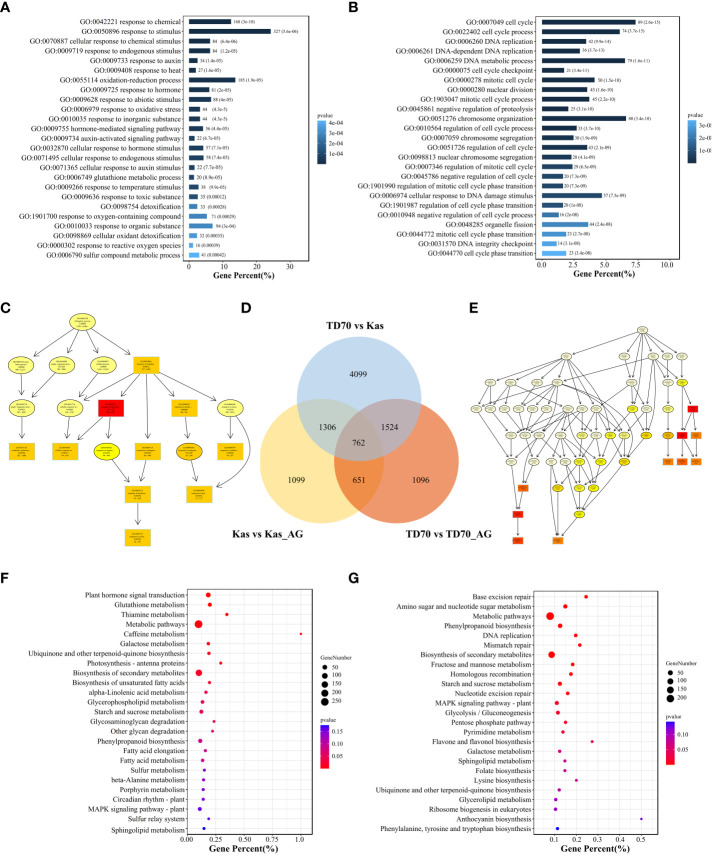
Transcriptome analysis of TD70 and Kasalath with/without AG treatment. **(A)** GO enrichment plot of TD70-specific DEGs response to AG treatment. **(B)** GO enrichment plot of Kasalath-specific DEGs response to AG treatment. **(C)** Biological process graphic result of TD70-specific DEGs. **(D)** Venn plot of DEGs among three groups: TD70 vs Kas, Kas vs Kas_AG and TD70 vs TD70_AG. **(C, E)** Biological process graphic result of Kasalath-specific DEGs. **(F)** KEGG enrichment plot of TD70-specific DEGs response to AG treatment. **(G)** KEGG enrichment plot of Kasalath-specific DEGs response to AG treatment.

For identifying candidate genes associated with AG traits, we further investigated transcriptome response during the process of tolerance to anaerobic germination. As a result, 2,405 DEGs from kasalath under AG treatment were identified and consider as Kas-specific DEGs response to AG. Similarly, 2,620 TD70-specific AG-responsive genes were also identified (**Figure 5D**). GO and KEGG enrichment were further employed for dissecting the potential molecular mechanism under AG treatment. From GO/KEGG enrichment results, amount of DEGs were found to be invovled in many biological processes such as “response to oxidative stress, GO:0006979”, “hormone-medidated signaling pathway, GO:0009755”, “detoxification, GO:0098754”, “Amino sugar and nucleotide sugar metabolism” (**Figure 5**). Combined with 12 stable QTLs, 103 expressed genes were located in QTL regions, of which 31 genes such as *OsUsp1*, *OsMADS4*, *PR5*, *OsSPARK4*, *OsSUAR56*, *TOND1* show significant among different groups (**Supplementary Table S6**).

## Discussion

### Phenotype identification of rice seed AG tolerance

Rice varieties with strong tolerance to anaerobic germination (AG) are suitable for direct sowing. So far, there are two methods to identify rice AG tolerance. One is according to the survival rate of 10-20 cm under submerged treatment for 21 days, which has been used to identify several QTLs (Angaji et al., 2010). However, this method is time-consuming and laborious. Another is to measure the coleoptile length. The QTL associated with coleoptile length coincided with that detected by using survival rate (Hsu and Tung, 2015). In order to study rice AG tolerance more comprehensively, four traits (CL, CD, CSA, CV) relevant to coleoptile were analyzed in this study.

Firstly, we identified the coleoptile phenotype of two parental materials under anaerobic treatment. The result showed that Kasalath germinated earlier than TD70, and the coleoptile grew rapidly. On the sixth day of treatment, the length, surface area and volume of Kasalath coleoptile were significantly higher than TD70 (but coleoptile diameter), which indicated that Kasalath had stronger AG tolerance than TD70 (**Figure 1**). Further analysis of the four traits of coleoptile in RIL populations under AG conditions showed that they segregated into various degrees, which showed the characteristics of normal distribution. Therefore, the result indicated it was possible to use Kasalath and TD70 construction population to locate QTL of AG tolerance.

### Joint analysis using the high-density genetic map and RNA-Seq

Recently, the next-generation sequencing technology (NGS) has been used for population genotyping, which is improving the accuracy of QTL mapping (Huang et al., 2009; Wang et al., 2011). In this study, we sequenced 186 RILs and detected 1,344,770 high-quality population SNPs with uniform distribution in the whole genome. The recombination breakpoint was determined by checking the position of genotype change. This method transformed the original SNP markers into effective recombination bins, which could be considered an effective genetic marker (Wang et al., 2011). With this method, a high-density genetic map containing 12,328 bin markers was constructed in this study, and the mapping of QTLs was conducted using this map.

In this study, we measured four coleoptile traits, CL, CD, CSA and CV, on the fourth and sixth days of anaerobic treatment. The results showed that the phenotypic variations of CL, CD, CSA and CV were normal distribution in RILs, indicating that they were consistent with the characteristics of quantitative genetics controlled by multiple genes (**Figure 2**). In this population, 50 QTLs associated with coleoptile traits under the anaerobic were identified, and 12 stable loci, including 20 QTLs, were obtained through further analysis.

Hypoxia under anaerobic conditions changes plant metabolism, thus affecting plant growth. The change in the glycolysis pathway is one of the typical metabolomic responses to hypoxia. Upregulation of amylase, trehalose-6-phosphate phosphatase, hexokinase and other enzyme activities under hypoxia conditions provided sufficient evidence (Magneschi and Perata, 2009; Kretzschmar et al., 2015; Loreti et al., 2018). When the oxygen concentration is low, carbohydrate metabolism is subsequently inhibited, especially the step of starch decomposition into monosaccharides for glycolysis (Miro and Ismail, 2013). These studies identified complex mechanisms related to coleoptile growth, including carbohydrate metabolism, fermentation, hormone induction, cell division and expansion. Many transcriptome analyses are conducted to explore the molecular mechanism in regulating rice coleoptile growth under hypoxia and anaerobic conditions. The results showed that starch degradation and fermentation-related genes were up-regulated in anoxic rice seedlings (Narsai et al., 2015; Hsu and Tung, 2017).

Research shows that the elongation of rice coleoptiles is relevant to the hexokinase gene (*HXK6*, LOC_Os01g53930) under anaerobic conditions (Hsu and Tung, 2015). LOC_ Os06g03520, a candidate gene encoding DUF domain protein, is highly expressed in the coleoptile under flooded conditions (Zhang et al., 2017). *OsTPP7* gene, encodes a trehalose-6-phosphate phosphatase. It indicates a low sugar utilization rate by increasing the turnover of trehalose-6-phosphate, which can also improve the pool capacity in heterotrophic tissue. Furthermore, it can enhance starch utilization, drive germinating embryo growth, prolong coleoptiles, and enhance anaerobic germination tolerance (Kretzschmar et al., 2015). AG tolerance can be achieved by combining the number of favorable alleles in different varieties.

### Improving AG tolerance by pyramiding breeding

Recently, OsGF14h, a key gene determining its strong germination and emergence under hypoxia, was cloned from japonica weedy rice *via* genome-wide associating study and QTL mapping. This gene encodes a “14-3-3 protein”, which is the upstream switch of abscisic acid (ABA) signal transduction. By interacting with two transcription factors, *OsHOX3* and *OsVP1*, the gene inhibits the activity of the ABA receptor *OsPYL5* in a hypoxic environment, reduces the sensitivity of ABA, and also activates the biosynthesis of gibberellin (GA), ultimately causing strong germination and emergence of weedy rice seeds. However, in modern genetic improvement, the lost of 4 bases of *OsGF14h* in cultivated *japonica* rice caused the the weakening of its function, so germination and emergence were limited under hypoxia conditions (Sun et al., 2022).

## Conclusion

In this study, a high-density genetic map was constructed by whole genome resequencing to identify QTL for CL, CD, CSA and CV of the anaerobically treated coleoptile. A total of 42 loci were identified on 4 and 6 days of treatment. A total of 12 stable loci were identified, of which 6 coincided with previously reported loci. The localization results were further validated by verifying that the pyramiding of 12 QTLs could improve AG tolerance in rice in a population. We performed transcriptome sequencing analysis of three critical stages of germination in parental AG, and combined the mapping results of 12 QTLs to obtain 275 possible candidate genes. These results further elucidated the genetic mechanism of AG tolerance in rice seeds.

## Data availability statement

The data presented in the study are deposited in the SRA repository, accession number PRJNA896343.

## Author contributions

YZ and YL conceived and designed the study. WL, HD, BP, JC, BH, FH, and YL performed the QTL mapping and RNAseq analysis. WL constructed the NILs, performed phenotypic analysis. WL and YL wrote the manuscript. All authors contributed to the article and approved the submitted version.
